# Between Adaptation and Resistance: A Study on Resilience Competencies, Stress, and Well-Being in German VET Teachers

**DOI:** 10.3389/fpsyg.2021.619912

**Published:** 2021-07-06

**Authors:** Tobias Kärner, Matthias Bottling, Edgar Friederichs, Detlef Sembill

**Affiliations:** ^1^Economic and Business Education (560A), University of Hohenheim, Stuttgart, Germany; ^2^Centre for Learning and Development and Honorary Professorship for Economic and Business Education, University of Bamberg, Bamberg, Germany; ^3^Economic and Business Education, University of Bamberg, Bamberg, Germany

**Keywords:** occupational stress, cluster analysis, well-being, vocational education and training, resilience

## Abstract

We demonstrate the relationships between occupational demands in German vocational education and training (VET) teacher training, stress symptoms, and different behavioral resilience competencies. Taking into account interindividual differences in resilience competencies, we use a typological approach to identify different types of (trainee) teachers classified by their degrees and configurations of resilience competencies. Our empirical analysis is based on questionnaire data from 131 German vocational trainees and qualified teachers. The results reveal, among other things, that all three resilience competencies—resistance, flexibility, and dynamism—are significantly negatively correlated with the demands of working conditions and workload. Via a latent class analysis, we were able to identify three groups of (trainee) teachers who differed in their resilience competencies to adapt appropriately to different situations and their requirements (“behavioral flexibility”), to recover rapidly from setbacks and to defy the expectations of others (“behavioral resistance”), and to initiate changes as soon as they are necessary or desirable (“behavioral dynamics”). More resilient (trainee) teachers show, among other things, lower values for anxiety as an emotional stress symptom and higher values for job engagement. The findings are discussed with regard to implications for VET teacher training and we stress the need for equilibration on a systemic perspective.

## Introduction

Today, mental health problems are becoming more and more prevalent in Western society. According to Wittchen et al. (2011, p. 656), “in every year over a third of the total EU population suffers from mental disorders. The true size of “disorders of the brain” including neurological disorders is even considerably larger.” One of the main causes for this phenomenon is stress, which is often a result of time and social pressure and pressure to adapt (Sembill, 2015). Johnson et al. (2005) have shown that work-related stress differs considerably in various occupations. Teachers, especially, tend to show worse than average levels in physical health, psychological well-being, and job satisfaction compared to other occupations (Kieschke and Schaarschmidt, 2008; Paulus and Schumacher, 2008). Numerous studies confirm that the teaching profession is associated with great stress (e.g., Pithers and Fogarty, 1995; Kyriacou, 2001; Montgomery and Rupp, 2005; Zurlo et al., 2007; Stoeber and Rennert, 2008; Liu and Onwuegbuzie, 2012; Newberry and Allsop, 2017). Not only experienced teachers but also prospective teachers suffer from work-related stress that can already be observed in the teachers' early career when they start to work as beginning teachers or even earlier as trainees (Chaplain, 2008; Gardner, 2010; Harmsen et al., 2018).

The preparatory teacher traineeship in Germany shapes many prospective teachers for their subsequent career. Studies have shown that many trainee teachers associate this preparatory traineeship with heavy burdens and stressful experiences and as a time full of pressure to adapt, ambivalences and conflict potential (e.g., Christ, 2004; Speck et al., 2007; Klusmann et al., 2012; Schumann, 2019). Trainee teachers have to withstand a variety of potential stressors, including, among other things, high workload and performance pressure, conflicts with students or colleagues, or the dependency on the instructors (e.g., Kyriacou and Stephens, 1999; Christ et al., 2004; Chaplain, 2008). Nevertheless, some trainee teachers seem to cope better with the requirements than others (Englert et al., 2006; Chaplain, 2008; Košinár, 2014). One possible explanation for this phenomenon can be assigned to the concept of *resilience*, which refers “to a dynamic process encompassing positive adaptation within the context of significant adversity” (Luthar et al., 2000, p. 543). As the construct of resilience is very complex, it has been discussed and measured in the past 20 years in multiple ways [for an overview, see for example, Beltman et al. (2011)]. In this study, we refer to three *resilience competencies* (*flexibility, dynamism*, and *resistance*) that are indicative of resilient behaviors (Friederichs et al., 2019, 2021). As typological approaches are common in educational research or psychological science (e.g., Hayenga and Corpus, 2010; Boiché and Stephan, 2014; Martinent and Decret, 2015; Sappa et al., 2018), they also seem to be promising in the analysis of teacher resilience from a person-centered view. Thus, taking into account interindividual differences in sets of resilient behavioral competencies may provide a possible explanation why (prospective) teachers differ in their perception of work-related stressors and in their psychological and physiological stress symptoms. Further, groupings according to resilience profiles could reveal that different groups of teachers need different types of support, such as mentoring or other kinds of interventions.

The study of teacher resilience is certainly not a new field of research. However, the focus on vocational education and training (VET) teachers, especially during teacher training, represents a missing field that has not yet been considered sufficiently (with the exception of Sappa and colleagues). Consequently, our primary interest can be formulated as the following research question: How do VET (trainee) teachers differ in terms of their resilience competencies? We are also interested in how those groups perceive, on the one hand, *occupational demands* (in terms of all objectively seen requirements made upon teachers that result from the job) and, on the other hand, differ with regard to *subjective appraisal* of experienced demands and various *stress symptoms*.

## Theoretical Framework

The first part of this section introduces the German context of VET teacher training, as teacher training in Germany generally differs from teacher training in other countries. The following section then presents the underlying model of our study, which has its origin in the model of teacher stress from Kyriacou and Sutcliffe (1978). The model basically consists of three components: (1) potential occupational stressors, which are appraised either as irrelevant, challenging, or threatening by a (trainee) teacher; (2) stress symptoms, which can be of a psychological or physiological nature; and (3) our construct of resilience, measured via the three behavioral resilience competencies flexibility, resistance, and dynamism.

### The Organizational Context of German VET Teacher Training

VET teacher training in Germany initially takes place at the university or college of education in the field of business or technical education; it includes a ten-week teaching practice, followed by a preparatory traineeship at the colleges of didactics and teacher education^1^, as well as directly in vocational schools (Deißinger and Kremer, 2004; Deißinger et al., 2018). Our study focuses on the so-called *second phase of teacher training*—the in-service training (Sembill, 1984)—in which the beginning VET teachers “complete […] a period of school-based and seminar-supported practical training focusing strongly on didactics and teaching competence (normally for 18–24 months, depending on the state)” (Deißinger et al., 2018, p. 34). Due to the federal structure, education policy in Germany is the responsibility of the federal states [BMBF (Federal Ministry of Education and Research), 2020]. As a result, the structures and contents of the preparatory traineeship differ among the federal states (Krüger, 2014). For example, the duration of the preparatory service for a career in higher education at vocational schools in the federal state of Baden-Wuerttemberg is 18 months, whereas the preparatory service for a teaching profession at vocational schools in the federal state of Bavaria lasts 24 months [KM Baden-Wuerttemberg (Ministry of Education, Youth and Sports Baden-Wuerttemberg), 2020; KM Bavaria (Bavarian State Ministry for Education Cultural Affairs), 2020]. Nevertheless, nationwide similarities can be identified. Normally, the traineeship takes place at two locations or learning venues: the colleges of didactics and teacher education and the training schools. The traineeship thus follows the principle of duality, similar to that of apprenticeship training in Germany, and offers the opportunity for “learning on the job” (Halász et al., 2004, p. 32). In most federal states, the traineeship consists of two phases: a preparatory phase that takes place exclusively at the colleges of didactics and teacher education and a second phase, in which the prospective teachers have to conduct a fixed amount of lessons independently (Bölting and Thomas, 2007).

### A Model of Teacher Stress

**Figure 1:**
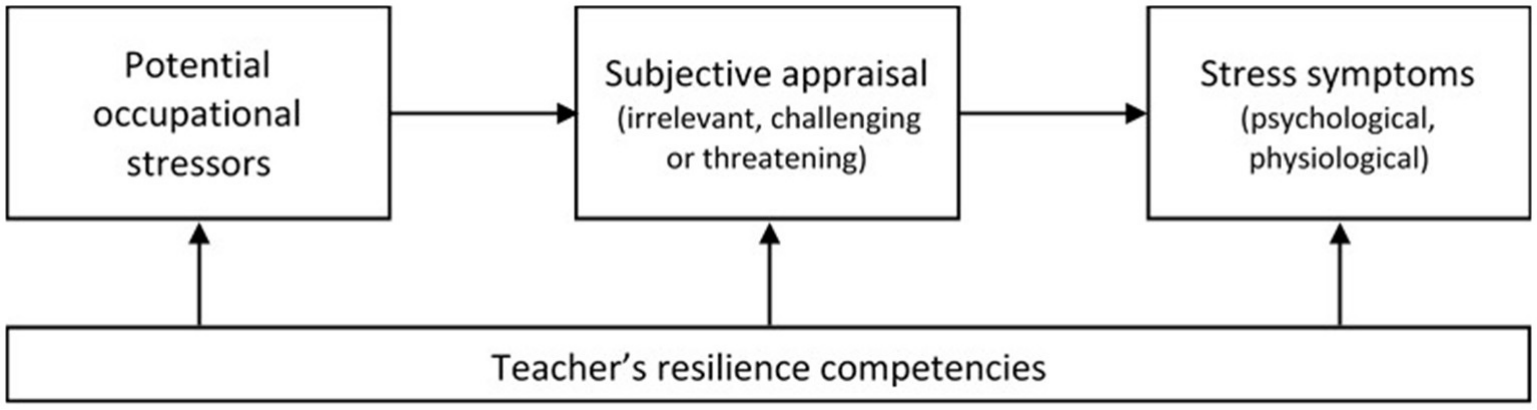
Model of teacher stress according to Kyriacou and Sutcliffe (1978).

There are different approaches for modeling stress that share the basic ideas of the transactional stress model of Lazarus and colleagues (Lazarus, 1966; Lazarus and Launier, 1981; Lazarus and Folkman, 1984; for an overview, see Van Dick and Stegmann, 2013). An established approach to describing stress in the teaching profession is the model of teacher stress from Kyriacou and Sutcliffe (1978, et passim), which also uses the theory of Lazarus and colleagues as an initial model that has proven itself in the past decades of research. The model is generic with respect to its basic psychological ideas. Even though the model of Kyriacou was first published in 1978, it was continuously used in psychological teacher stress research and applied in further applications (e.g., Kyriacou, 2001, 2011). We adapted the model of Kyriacou and Sutcliffe (1978, et passim) as shown in Figure 1.

The basic idea of the model is that strain can arise through the *subjective appraisal* of *potential occupational stressors* (see section Potential Stressors in VET Teacher Training). For instance, there might be teachers who perceive interaction with the students' parents as a threat, whereas others might appraise these interactions as irrelevant or even (positively) challenging. If a potential stressor is evaluated as a threat and the associated perceived strain exceeds the available resources, a subjectively perceived burden becomes stress, which can manifest itself in psychological (e.g., anxiety, anger, poor well-being) or physiological (e.g., headaches, nausea) *stress symptoms* (Kyriacou and Sutcliffe, 1978; Lazarus and Folkman, 1984; Kalisch et al., 2015; see section Stress Symptoms, Well-Being, and Work Experience). Thus, objectively identical potential occupational stressors can be perceived as differently burdensome depending on the individuals' resources (Sembill, 2012). How individuals actually cope with stressful encounters mainly depends on the *resources* that are available to them and the constraints that inhibit use of these resources in the context of the specific encounter (Lazarus and Folkman, 1984). Here, *resilience* is seen as an ability to meet or cope with occupational demands and can therefore be regarded as one of the teachers' coping resources (see section Three Resilience Competencies: Dynamism, Flexibility, and Resistance). In the following and in reference to the model of teacher stress according to Kyriacou and Sutcliffe (1978, et passim), we will describe potential stressors in VET teacher training, different stress symptoms, and different competencies of teacher resilience in greater detail.

#### Potential Stressors in VET Teacher Training

Numerous studies have already explored the sources of work-related teacher stress (e.g., Pithers and Fogarty, 1995; Griffith et al., 1999; Zurlo et al., 2007). There are also studies that have explored the sources of stress among prospective teachers (e.g., Chaplain, 2008; Gardner, 2010; Klusmann et al., 2012). As Kyriacou (2001) points out, identified sources of (prospective) teacher stress must be viewed in the context of a teacher's perception, individual characteristics, and their environment. Depending on the country in which a teacher works, even country-specific main sources of teacher stress are possible. Therefore, the term “potential stressor” seemed to be the most appropriate to us.

Christ (2004), Rudow (2000), and Van Dick (1999) categorized potential stressors in the teaching profession and teacher education in general. Specific VET-related stress factors are described by Sappa and colleagues (Sappa et al., 2015, 2019, 2021; Boldrini et al., 2019) and Kärner and colleagues (Kärner et al., 2016; Kärner and Höning, 2021). In that regard, stressors reported by VET teachers are, among other things, high workload and pressure to perform, disturbances of the regular workflow, time pressure and deadlines, social conflicts in the workplace, and additional administrative tasks (Kärner et al., 2016). Further, VET teachers suffer from the decline of the social reputation of vocational teaching and learning, students' lack of professional motivation, maturity and engagement, and the heterogeneity of students' prior knowledge and experiences [Sappa et al., 2021; see also Kärner and Höning (2021)].

**Figure 2:**
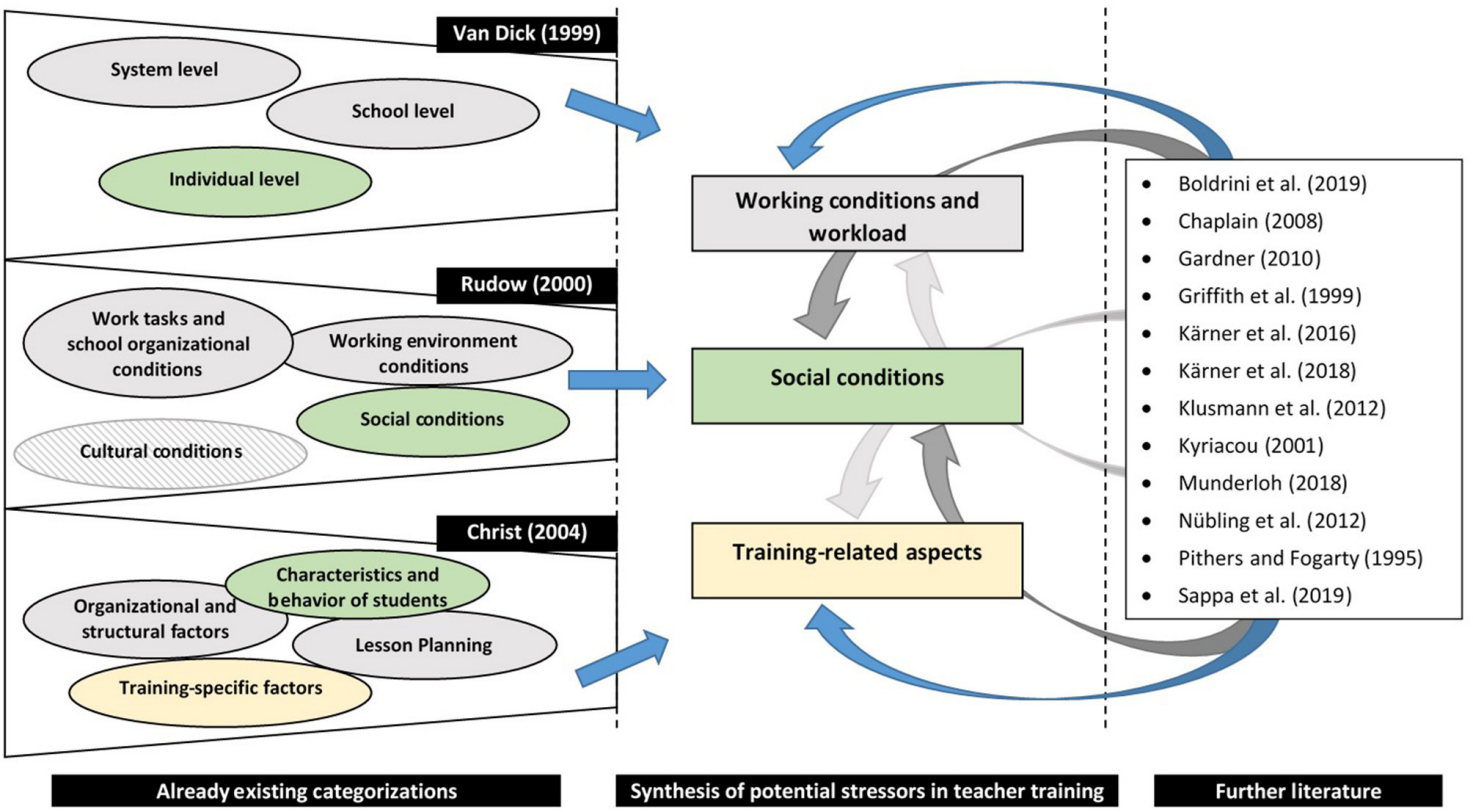
Three main sources of potential occupational stressors in VET teacher training.

By taking into account existing categorizations and reviewing the existing literature regarding the sources of work-related teacher stress, we derived three main sources of potential occupational stressors. The categories can be summarized under the umbrella terms *working conditions and workload* as well as *social conditions*. Only Christ (2004) refers to training-specific stressors, which are especially relevant during teacher training and thus, form our third category *training-related aspects*. Figure 2 illustrates our synthesis of potential stressors in teacher training.

The potential stress factor *working conditions and workload* describes occupational demands such as workload, time pressure or preparation and follow-up for classes. Especially in teacher training, coping with several demands from different institutions (e.g., seminar, school or mentor) may lead to stress. Additionally, the prospective teachers are under constant observation and may be affected in their private life as well (e.g., hobbies, partnership or friends), which is also known as “work-privacy conflict” (e.g., Kyriacou, 2001; Chaplain, 2008; Nübling et al., 2012; Kärner et al., 2016; Munderloh, 2018).

Stress also results from the typical *training-related aspects* of teacher training in Germany. This includes the relationship of trainee teachers with the mentor, seminar teacher or headmaster, dealing with colleagues and the ambivalent role of the seminar teacher, who is simultaneously coach and evaluator. Another potential stressor in this context could be the uncertainty about grading or performance evaluation (e.g., Christ, 2004; Klusmann et al., 2012; Kärner et al., 2018, 2019)^2^.

Specific *social conditions* are a third source of stress in teacher training. This aspect includes the student's behavior during classes, conflicts between students among themselves, but also conflicts between certain students and the trainee teacher. Great heterogeneity in one class is also experienced stressfully, as a trainee teacher has to cater to all students—with different learning styles and prior knowledge—at the same time. Dealing with parents also plays a significant role in this context because their expectations have to be met as well (e.g., Van Dick, 1999; Christ, 2004; Chaplain, 2008).

#### Stress Symptoms, Well-Being, and Work Experience

*Psychological stress symptoms* are responses such as anger, depression, or high job dissatisfaction (Kyriacou and Sutcliffe, 1978). Drüge et al. (2014) have compared psychosocial hazards and the resulting strains on trainee teachers with teachers and two other occupational groups. The analyses show that trainee teachers have appreciably higher values in burnout and cognitive stress symptoms and lower values in their health condition than the reference groups. Chaplain (2008), for instance, investigated psychological distress among trainee teachers in England and found female trainee teachers had, compared to male trainee teachers, higher dissatisfaction regarding their current mental state.

Mental well-being is closely related to psychological stress and may be negatively affected if potential stressors are perceived as a threat (Kyriacou and Sutcliffe, 1978; Lazarus and Folkman, 1984). Teachers' well-being is often investigated in combination with occupational stress and burnout (Spilt et al., 2011). In that regard, *emotional exhaustion* is seen as the key symptom of burnout as a stress-related disorder (e.g., Klusmann et al., 2012), which results “from occupational stress among human service workers, including teachers” (Jennett et al., 2003, p. 583). Klusmann et al. (2012) found that the emotional exhaustion reported by trainee teachers is, on average, comparable to that of teachers who have been in the profession for an average of 20 years. As the study of Christ et al. (2004) on trainee teachers shows, a higher level of perceived burden is associated with poorer psychological well-being and increased physical complaints. Lesson- and training-related burden factors appear to be especially highly correlated with well-being and physical symptoms.

Besides psychological symptoms, stress manifests itself in *physiological symptoms*, which are characterized, for example, by high blood pressure (Kyriacou and Sutcliffe, 1978) or other physical symptoms such as headaches or nausea. Lazarus and Folkman (1984) defined this kind of response correlate as *somatic health* and distinguish between short-term and long-term adaptational health outcomes.

According to Affolter (2019), in teacher stress research positive work experience has also become important in recent years. As a counterpart to negative concepts, such as stress, strain or burnout that can result from negative work experience, this positive perspective can be divided into a short-term and long-term dimension. The concept of *job satisfaction* reflects the short-term dimension, whereas *work engagement* reflects the long-term dimension. Job satisfaction can be understood as a short-term positive response to the perception of several job characteristics, taking into account individual needs (Ulich and Wülser, 2004). Schaufeli and Bakker (2004b, p. 295) define “engagement as a positive, fulfilling, work-related state of mind that is characterized by vigor, dedication, and absorption.”

After describing potential stressors in VET teacher training as well as possible stress responses, we will now look at the characteristics of teachers' resilience that help prospective teachers to cope with stressful encounters and occupational demands.

#### Three Resilience Competencies: Dynamism, Flexibility, and Resistance

The concept of resilience was initially applied to the long-term development of children exposed to various risk factors (e.g., poverty or unstable family relations; see Werner and Smith, 1982) and, more generally, to the developmental psychopathology field in early childhood education and in childhood and adolescence (see Garmezy et al., 1984; Garmezy, 1985; Scheithauer et al., 2000). Due to the increasing relevance of mental health disorders, resilience research has consequently been extended to adulthood (e.g., Friederichs et al., 2019, 2021). Although there are striking parallels between the resilience in childhood and resilience in adulthood, Bonanno (2004, 2005) points out that adults have more resilience-promoting factors available when it comes to, for example, potentially traumatic events. In the context of (trainee) teachers, Gu and Day (2013, p. 26) have specified that teacher resilience “is not primarily associated with the capacity to “bounce back” or recover from highly traumatic experiences and events but, rather, the capacity to maintain equilibrium and a sense of commitment and agency in the everyday worlds in which teachers teach.” In the literature, the understanding and consequently the definition of the term resilience differ. Masten and Obradovic (2008, p. 2) define resilience generally as follows:

Individual resilience refers to the processes of, capacity for, or patterns of positive adaption during or following exposure to adverse experiences that have the potential to disrupt or destroy the successful functioning or development of the person.

Resilience is often also seen as psychological resistance, in the sense of it being a relatively stable personality trait. Here it is assumed that resilience is a given quantity that some individuals possess and others do not. However, this approach excludes the elucidation of the underlying behavioral processes of resilience and suggests that resilience-promoting intervention strategies are of little use (Bengel and Lyssenko, 2012).

The present study does not consider resilience as *one* stable personality trait but rather as a set of behavioral competencies that are required in a particular context to cope adequately with stress and to remain mentally and physically healthy (Friederichs et al., 2019, 2021). If we refer to the term *resilience competencies*, we somehow follow the idea from Howard and Johnson (2004, p. 403), who argue that “protective factors […] support “resilient” teachers” and are learnable. Resilience competencies can be defined as abilities to cope with occupational demands and to protect (trainee) teachers' health. Our understanding of resilience is based on Friederichs et al. (2019), who consider resilience from a multifactorial behavioral perspective. The underlying model is supported by the two-process model of behavior regulation according to Brandtstädter (2011; see also Brandtstädter and Rothermund, 2002; Brandtstädter, 2009), who distinguishes between *assimilative* and *accommodative* processes as part of a theoretical framework for coping processes. Assimilative processes strive to change given development and living conditions in favor of one's own objectives, while accommodative processes facilitate the adaption of personal goals to the possibilities or limitations of action. Additionally, Brandtstädter and Greve (1994) propose a third process, *immunizing*, in this context, which Leipold and Greve (2009) call *defensive mode*. Leipold and Greve (2009) describe this third reaction mode as a defensive process, in which the individual completely ignores and denies the meaning or existence of a problem. Dealing with problems in this case occurs completely in the background of the individual, and neither adaption to a problem nor active problem-solving takes place. Based on the mentioned postulated psychological processes of behavior regulation, the resilience model of Friederichs et al. (2019) assumes that the following three behavioral competencies of resilience form an individual's ability to cope with actual demands:

- *Behavioral flexibility* is understood as a competence of adaption—that is, to react appropriately to different situations and their requirements. It is the ability to accept other people and their peculiarities and, if necessary, to put one's own needs aside.
- *Behavioral dynamics* in terms of openness to change is seen as a competence to initiate changes as soon as they are necessary or desirable and to abandon time-honored habits or practices.
- *Behavioral resistance* in terms of persistence tendency is the competence to recover rapidly from setbacks and to defy the expectations of others—that is, to demarcate from others.

The three resilience competencies under consideration reflect individual evaluation and balancing processes between perceived expectations and expectation fulfillment as well as between own needs and external demands (Sembill and Kärner, 2018). It is assumed that the three behavioral competencies of resilience have a dynamic relationship with each other. Behavioral flexibility thus describes the tendency to react to external requirements and to put one's own interests and goals last. The tendency to persist can be seen as the opposite pole, since it involves distancing oneself from external expectations while at the same time fixating on one's own needs and goals. An extreme expression on one of the two poles is assumed to be maladaptive in the long run. Openness to change can be seen here as an equilibration regulative for being able to move appropriately on the continuum between adaptation and resistance (Sembill and Kärner, 2018; Friederichs et al., 2021).

In a previous study of Friederichs et al. (2021), the three mentioned resilience competencies were validated on a sample of 150 employees at a German automotive supplier company, and results showed that the resilience competence “dynamism” is significantly associated with parasympathetic parameters of heart rate variability that indicates somatic adaptation to stress. Further, all three resilience competencies are negatively correlated with different scales of the “Trier Inventory of Chronic Stress” (Schulz et al., 2004).

## Research Aim

By taking up the complex and even stressful working environment of (trainee) teachers, we are interested in identifying different types of (trainee) teachers classified by their degrees of resilience competencies. We therefore want to answer the following research question: How do VET (trainee) teachers differ in terms of their resilience competencies? To answer this general question and to examine (trainee) teachers' perception of occupational demands and stress symptoms, we derived the following hypotheses from our underlying model of teacher stress and reviewed literature:

*1) Different patterns, in terms of the three resilience competencies flexibility, resistance, and dynamism, can be identified among VET (trainee) teachers*. I.e., VET (trainee) teachers can be assigned to a group with a certain degree of resilient behavior, for example, a high/low degree of resilient behavior.
*2) The resilience competencies are significantly negatively correlated with perceived occupational demands and stress appraisal*.
*3) The resilience competencies are significantly negatively correlated with stress symptoms and emotional exhaustion and significantly positively correlated with work experience and well-being*.

As the research of resilience has focused in the past primarily on teachers in general and even less on teacher training, we wanted to widen this promising concept to the VET sector. At this point, a typological approach seems to be the means of choice for understanding why different (trainee) teachers perceive occupational demands differently and show different degrees of stress symptoms, although they are exposed to comparable structural settings.

## Methods

### Data Collection and Sample

Data were collected via an online questionnaire, which was provided via teacher forums and mailing lists. The study was conducted between December 2, 2019 and January 8, 2020. There were 845 clicks on the online questionnaire, and after data cleaning, we could use data from 131 VET (trainee) teachers who finished the relevant items, resulting in a response rate of 15.50%. Our sample consisted of 74 females and 57 males, with a mean age of 38.37 years (SD = 9.97, Min. = 24, Max. = 63). At the time of the survey, 74.8% of the sample had already completed their practical teacher training^3^, with a mean vocational experience as a teacher of 11.13 years (SD = 9.08, Min. = 1, Max. = 39); 84% of the sample (*n* = 110) were vocational (trainee) teachers in the subject area “business economics,” six (trainee) teachers (4.6%) worked in the field of technical education, eight (trainee) teachers (6.1%) worked in the subject area “household and socio-pedagogical” and seven (5.3%) participants taught in other domains (e.g., computer sciences, textile technology). All participants were informed about data protection compliance and provided written informed consent.

### Measures

Measures of resilience competencies, occupational demands and subjective stress appraisal, and stress symptoms, well-being and work experience are summarized in Table 1, including Cronbach's α as a measure for test-score reliability, the number of items per scale, an example item, and the reference literature. Measures of occupational demands and stress appraisal are based on our synthesis of potential stressors in teacher training. Thus, the potential stressors for trainee or experienced teachers reported in the original sources (Figure 2) were the basis for our item formulation. Further, measures of occupational demands and stress appraisal were adapted from the original sources concerning the introductory texts (“During teacher training, I experienced …”) as well as concerning scale ranges and evaluation modes (demand assessment and subjective appraisal). Measures of stress symptoms, well-being, and work experience were adapted from the original sources concerning the introductory texts (“During teacher training, …”) and scale ranges.

**Table 1:** Measures and operationalisation.

**Variables**	**Cronbach's α**	**No. of items**	**Example**	**References**
**Resilience competencies**				
Dynamism	0.849	4	I see changes as a chance for personal growth	Friederichs et al. (2019)
Resistance	0.629	4	Others cannot easily influence me	
Flexibility	0.675	4	I can accept decisions even though I do not like them	
**Occupational demands and stress appraisal**			During teacher training, I experienced …	
Working conditions and workload (demands)	0.874	6	... The workload (for instance because of many different tasks to manage) as …	Adapted from Van Dick (1999), Rudow (2000), Christ (2004), Christ et al. (2004), Kärner et al. (2016), Boldrini et al. (2019), and Kärner et al. (2019) (see also Figure 2)
Working conditions and workload (appraisal)	0.826	6		
Social conditions (demands)	0.711	5	... Conflicts between me and some students as …	
Social conditions (appraisal)	0.798	5		
Training-related aspects (demands)	0.744	3	... The ambivalent role of my instructor (coach at the one hand, evaluator on the other hand) as …	
Training-related aspects (appraisal)	0.708	3		
**Stress symptoms, well-being, and work experience**			During teacher training, …	
Physical stress symptoms	0.820	6	... I suffered from headaches	Adapted from Lohaus et al. (2018)
Anger	0.861	4	... I was angry	
Anxiety	0.859	4	... I was nervous	
Well-being	0.915	4	... I was happy	
Emotional exhaustion	0.858	4	... I felt emotional exhausted	Adapted from Maslach and Jackson (1981) and Barth (1985)
Job satisfaction	0.903	5	… I was delighted in what I was doing	Adapted from Westermann et al. (1996), Maes and Van der Doef (1997), Sann (2003), and Affolter (2019)
Job engagement	0.891	9	... I was overflowing with energy	Adapted from Schaufeli and Bakker (2004a)

The three *resilience scales* were assessed via a 5-point Likert-type scale (1 = “strongly disagree,” 2 = “disagree,” 3 = “neither agree nor disagree,” 4 = “agree,” and 5= “strongly agree”).

For analyzing participants' perception of *occupational demands* during teacher training, the participants were asked about how demanding they experienced the named demands to be on a 4-point Likert-type scale (1 = “not demanding,” 2 = “slightly demanding,” 3 = “demanding,” and 4 = “very demanding”). Additionally, *subjective appraisal* of experienced demands was assessed, and participants were asked whether they perceived the demands as irrelevant (= 1), challenging (= 2) or threatening (= 3).

Concerning *stress symptoms*, participants were asked how often they experienced physical stress symptoms, emotional stress symptoms, well-being, and emotional exhaustion during teacher training (5-point Likert-type scale: 1 = “never,” 2 = “seldom,” 3 = “sometimes,” 4 = “often,” and 5 = “very often” for the stress symptoms). Furthermore, participants were asked how strongly they were *satisfied* and *engaged* in the job (4-point Likert-type scale: 1 = “strongly disagree,” 2 = “disagree,” 3 = “agree,” and 4 = “strongly agree” for job satisfaction and job engagement).

Cronbach's α values indicated sufficient reliability for measurements.

### Statistical Analysis

To assess interindividual differences in behavioral resilience competencies, we used a latent class analysis (LCA) as a statistical method to identify homogenous subgroups in a sample. In the current study, the classification into different classes was conducted on the basis of the mean scales for the three resilience scales. In order to determine the most adequate number of classes, different solutions are compared on the basis of common indices (Hagenaars and McCutcheon, 2002). For cluster identification we used Mplus 8 (Muthén and Muthén, 1998–2017). For analyzing descriptive data, Pearson correlations between variables, and cluster differences via ANOVAs, we used IBM SPSS 26.

## Findings

### Descriptive Data and Pearson Correlations

Table 2 contains descriptive data and Pearson correlation coefficients. Correlations show small but significant positive associates between the three *resilience competencies*. The three resilience scales are negatively correlated with experienced demands concerning working conditions and workload, as well as with subjective appraisal. This means that the higher the values of the three resilience competencies are, the less working conditions and workload are experienced as demanding and the less the stressors in question are perceived as threatening. Furthermore, “flexibility” is significantly negatively correlated with experienced demands concerning social conditions and training-related aspects. It is also significantly negatively correlated with anger, anxiety, and emotional exhaustion and significantly positively correlated with well-being, job satisfaction, and job engagement. The resilience scale “dynamism” is significantly negatively correlated with anxiety and emotional exhaustion and significantly positively correlated with job satisfaction and job engagement. None of the resilience scales are significantly correlated with sociodemographic data.

**Table 2:** Descriptive data and Pearson correlations.

**Variables**		**Min**.	**Max**.	**M**	**SD**	**1**.	**2**.	**3**.	**4**.	**5**.	**6**.	**7**.	**8**.	**9**.	**10**.	**11**.	**12**.	**13**.	**14**.	**15**.	**16**.	**17**.	**18**.	**19**.	**20**.
	**Resilience competencies**																								
1.	Dynamism	2.75	5.00	4.03	0.62																				
2.	Resistance	1.75	4.50	2.93	0.63	0.20^*^																			
3.	Flexibility	1.75	4.75	3.33	0.65	0.22^*^	0.19^*^																		
	**Occupational demands and stress appraisal**																								
4.	Working conditions and workload (demands)	1.00	4.00	3.01	0.65	−0.20^*^	−0.32^**^	−0.21^*^																	
5.	Working conditions and workload (appraisal)	1.00	3.00	2.15	0.44	−0.15	−0.35^***^	−0.21^*^	0.74^***^																
6.	Social conditions (demands)	1.00	3.20	2.04	0.47	−0.16	−0.11	−0.23^*^	0.32^***^	0.19^*^															
7.	Social conditions (appraisal)	1.00	2.80	1.71	0.38	0.04	−0.14	0.05	0.11	0.36^***^	0.40^***^														
8.	Training-related aspects (demands)	1.00	4.00	2.26	0.70	−0.06	0.01	−0.20^*^	0.38^***^	0.26^**^	0.23^*^	−0.03													
9.	Training-related aspects (appraisal)	1.00	3.00	1.76	0.52	−0.06	−0.15	−0.04	0.31^**^	0.41^***^	0.09	0.36^***^	0.61^***^												
	**Stress symptoms and work experience**																								
10.	Physical stress symptoms	1.00	4.33	1.90	0.72	−0.11	−0.11	−0.14	0.47^***^	0.40^***^	0.25^**^	0.00	0.16	0.04											
11.	Anger	1.00	5.00	2.59	0.86	0.03	0.12	−0.26^**^	0.41^***^	0.25^**^	0.16	−0.10	0.43^***^	0.23^**^	0.41^***^										
12.	Anxiety	1.25	5.00	3.41	0.87	−0.32^***^	−0.17	−0.21^*^	0.57^***^	0.48^***^	0.34^***^	−0.01	0.28^**^	0.19^*^	0.64^***^	0.45^***^									
13.	Well-being	1.50	5.00	3.53	0.73	0.13	0.09	0.30^**^	−0.51^***^	−0.42^***^	−0.08	0.04	−0.41^***^	−0.20^*^	−0.45^***^	−0.49^***^	−0.54^***^								
14.	Emotional exhaustion	1.00	5.00	3.25	0.93	−0.20^*^	−0.18	−0.24^**^	0.78^***^	0.64^***^	0.22^*^	−0.01	0.34^***^	0.19	0.56^***^	0.52^***^	0.68^***^	−0.62^***^							
15.	Job satisfaction	1.20	4.00	3.06	0.70	0.22^*^	0.05	0.19^*^	−0.49^***^	−0.29^**^	−0.16	0.02	−0.27^**^	−0.02	−0.34^***^	−0.40^***^	−0.49^***^	0.69^***^	−0.55^***^						
16.	Job engagement	1.56	4.00	2.88	0.56	0.28^**^	0.04	0.21^*^	−0.47^***^	−0.33^***^	−0.16	−0.03	−0.27^**^	−0.10	−0.40^***^	−0.43^***^	−0.52^***^	0.70^***^	−0.56^***^	0.88^***^					
	**Sociodemographic variables**																								
17.	Gender^a^ (% female)	56.50				−0.15	0.16	−0.01	−0.22^*^	−0.17	−0.08	0.01	−0.04	−0.03	−0.25^**^	−0.11	−0.13	0.05	−0.19^*^	0.10	0.06				
18.	Age	24	63	38.37	9.97	0.12	0.16	−0.01	−0.16	−0.14	0.19^*^	0.14	0.09	−0.02	−0.24^**^	−0.10	−0.19^*^	0.07	−0.24^**^	0.02	0.06	−0.01			
19.	Training status^b^ (% completed)	74.80				0.12	0.05	−0.04	−0.29^**^	−0.19^*^	0.11	0.21^*^	0.06	0.08	−0.20^*^	−0.11	−0.19^*^	0.14	−0.33^***^	0.16	0.19^*^	−0.02	0.53^***^		
20.	Time after training	1	39	11.13	9.08	0.07	0.04	0.04	−0.03	−0.04	0.19	0.09	0.01	−0.08	−0.12	−0.14	−0.11	0.11	−0.15	0.04	0.05	−0.03	0.88^***^	–	
21.	Final grade^c^	1.00	3.20	1.77	0.47	−0.07	0.12	−0.10	0.18	0.10	0.00	−0.16	0.39^***^	0.17	0.24^*^	0.37^***^	0.38^***^	−0.43^***^	0.29^**^	−0.42^***^	−0.44^***^	0.01	0.04	–	−0.03

* *p < 0.05*,

** *p < 0.01*,

*** *p < 0.001; 80 ≤ n ≤ 131;*

a *0 = female, 1 = male;*

b *0 = in teacher training, 1 = teacher training completed;*

c *1 = very good, 2 = good, 3 = satisfactory, 4 = sufficient, 5 = not sufficient/failed*.

*Working conditions and workload* (experienced demands and stress appraisal) are significantly positively correlated with physical and emotional stress symptoms (anger, anxiety) and emotional exhaustion, and the mentioned occupational demand is significantly negatively correlated with well-being, job satisfaction, and job engagement. Demands resulting from *social conditions* are significantly positively correlated with physical stress symptoms, anxiety, and emotional exhaustion. Demands resulting from *training-related aspects* are significantly positively correlated with emotional stress symptoms and emotional exhaustion, and they are significantly negatively correlated with well-being, job satisfaction, and job engagement.

### Cluster Identification and Cluster Differences

Table 3 contains information concerning cluster identification and model fit. Likelihood-ratio tests (VLMRT, aLMRT and PBLRT) showed that the 3-cluster solution fit best. Cluster 1 contains 31 (23.66%) participants, Cluster 2 contains 37 (28.24%), and Cluster 3 contains 63 (48.09%) participants.

**Table 3:** Cluster identification and model fit information.

		**No. of classes**				
		**1**	**2**	**3**	**4**	**5**
Cell frequencies per class	1	131	21	31	29	13
	2		110	37	64	16
	3			63	19	63
	4				19	17
	5					22
**Model fit information**						
No. of free parameters		6	10	14	18	22
LL		−375.771	−366.621	−355.446	−351.007	−346.084
AIC		763.542	753.242	738.892	738.015	736.169
BIC		780.793	781.994	779.145	789.768	799.423
ssaBIC		761.816	750.365	734.864	732.836	729.839
Entropy		NA^a^	0.799	0.867	0.821	0.826
VLMRT		NA^a^	0.001	0.039	0.170	0.589
aLMRT		NA^a^	0.002	0.047	0.187	0.604
PBLRT		NA^a^	<0.001	<0.001	0.333	0.308

*LL, Log likelihood; AIC, Akaike's Information Criterion; BIC, Bayesian Information Criterion; ssaBIC, Sample-size adjusted Bayesian Information Criterion; VLMRT, Vuong–Lo–Mendell–Rubin Likelihood-Ratio Test (p-value); aLMRT, Lo–Mendell–Rubin adjusted Likelihood-Ratio Test (p-value); PBLRT, Parametric Bootstrap-Likelihood-Ratio Test (p-value);*

a *not available for the one-class model*.

In addition to the model fit criteria, the mean class membership probabilities also indicate an acceptable 3-cluster solution (Table 4).

**Table 4:** Mean class membership probabilities.

	**Participants of Class 1**		**Participants of Class 2**		**Participants of Class 3**	
	***M***	**SE(M)**	***M***	**SE(M)**	***M***	**SE(M)**
MP for Class 1	0.905	0.029	0.000	0.000	0.013	0.008
MP for Class 2	0.000	0.000	0.928	0.022	0.029	0.009
MP for Class 3	0.095	0.029	0.072	0.022	0.958	0.012

*MP, Membership probabilities*.

**Figure 3:**
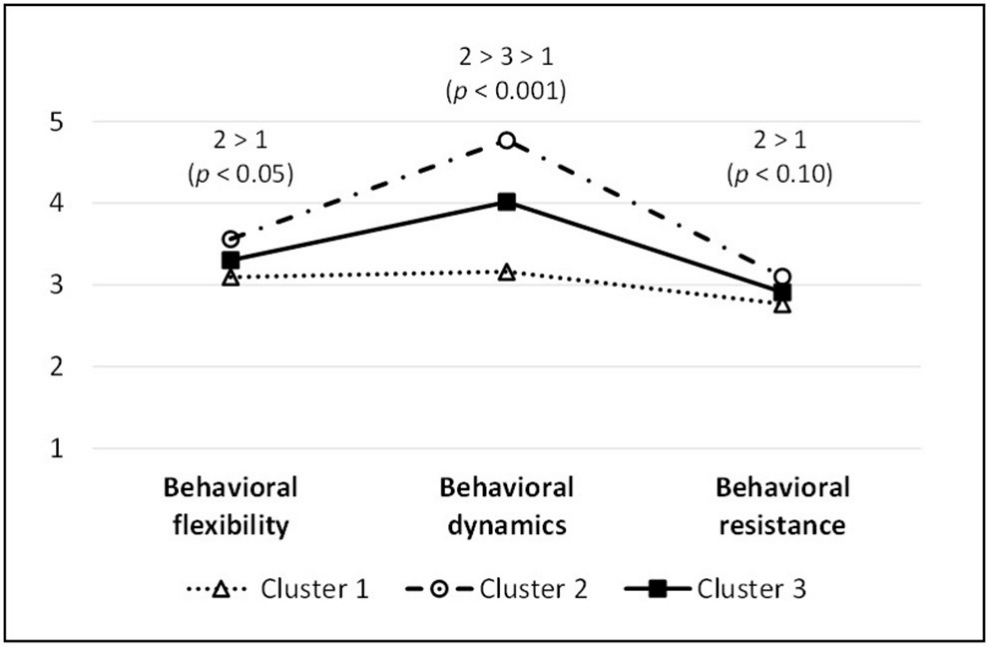
Cluster profiles.

Figure 3 shows cluster profiles concerning the three resilience competencies, and Table 5 shows cluster differences concerning all assessed variables. Participants in Cluster 2 show the highest values in “dynamism” (*p* < 0.001; large effect), “flexibility” (*p* < 0.05; medium effect), and “resistance” (*p* < 0.10; small effect), whereas participants in Cluster 1 show the lowest values in these resilience scales. Participants in Cluster 2, compared to participants in Cluster 1, also show the lowest values in demanding working conditions and workload (*p* < 0.05) as well as in demanding social conditions (*p* < 0.10). Concerning anxiety as an emotional stress symptom, participants in Cluster 2 show the lowest values, and they show higher values for job engagement compared to participants in Cluster 1. Furthermore, participants in Cluster 2 descriptively show the lowest scores on physical stress symptoms and emotional exhaustion, and the highest scores on job satisfaction (*p* < 0.10). There are no significant cluster differences in terms of age, time after training, and final grade, or concerning gender (*p* = 0.468) and training status (*p* = 0.450; via Chi-squared tests).

**Table 5:** Cluster differences (ANOVAs).

**Variables**	**Cluster 1**			**Cluster 2**			**Cluster 3**			***p***	**partial η^2^**	***Post-hoc*^**a**^**
	**M**	**SD**	***n***	**M**	**SD**	***n***	**M**	**SD**	***n***			
**Resilience competencies**													
Dynamism	3.16	0.25	31	4.77	0.19	37	4.02	0.20	63	<0.001	0.886	2 > 3 > 1
Resistance	2.77	0.51	31	3.10	0.72	37	2.91	0.62	63	0.087	0.037	
Flexibility	3.10	0.72	31	3.56	0.62	37	3.30	0.59	63	0.011	0.068	2 > 1
**Occupational demands and stress appraisal**													
Working conditions and workload (demands)	3.16	0.46	29	2.75	0.80	32	3.08	0.60	54	0.024	0.064	2 < 1
Working conditions and workload (appraisal)	2.22	0.29	29	2.01	0.51	31	2.20	0.45	52	0.104	0.041	
Social conditions (demands)	2.10	0.40	29	1.88	0.51	32	2.10	0.47	54	0.068	0.047	
Social conditions (appraisal)	1.64	0.37	29	1.71	0.46	31	1.74	0.34	52	0.478	0.013	
Training-related aspects (demands)	2.39	0.75	29	2.17	0.71	32	2.23	0.67	54	0.443	0.014	
Training-related aspects (appraisal)	1.81	0.52	28	1.68	0.55	31	1.78	0.51	52	0.588	0.010	
**Stress symptoms and work experience**													
Physical stress symptoms	1.94	0.62	29	1.65	0.55	32	2.02	0.83	56	0.063	0.047	
Anger	2.57	0.62	29	2.54	1.08	32	2.63	0.84	56	0.897	0.002	
Anxiety	3.82	0.73	29	2.95	0.77	32	3.47	0.87	56	<0.001	0.138	2 < 1, 2 < 3
Well-being	3.38	0.59	29	3.68	0.82	32	3.52	0.73	56	0.272	0.023	
Emotional exhaustion	3.49	0.81	29	2.96	1.00	32	3.29	0.91	56	0.074	0.045	
Job satisfaction	2.87	0.67	29	3.28	0.75	32	3.03	0.68	56	0.072	0.045	
Job engagement	2.71	0.50	29	3.12	0.57	32	2.83	0.55	56	0.010	0.078	2 > 1
**Personal information**													
Age	36.77	10.67	31	39.97	9.03	37	38.21	10.15	63	0.416	0.014	
Time after training	10.10	10.29	21	11.80	7.88	30	11.17	9.37	47	0.807	0.005	
Final grade^b^	1.81	0.37	21	1.72	0.57	30	1.78	0.44	47	0.805	0.005	

a *Post-hoc test with Bonferroni correction;*

b *5-point grading scale: 1 = very good, 2 = good, 3 = satisfactory, 4 = sufficient, 5 = not sufficient/failed*.

## Discussion

### Summary of Findings

This paper attempts to explain the differences between German VET (trainee) teachers' resilience competencies and their diverse perception of the demands and objective burdens made upon them. The concept of resilience was introduced as a set of competencies required to cope adequately with teacher stress and to remain mentally and physically healthy. To answer our research question, we derived three hypotheses.

Regarding our *first* hypothesis, we found three manifestations of resilience competencies in our sample, as the 3-cluster solution fit best. Cluster 2 (28.24%) indicated the strongest degree of behavioral resilience competencies, followed by Cluster 3 (48.09%) with an average level, and Cluster 1 (23.66%) with the lowest level of resilience competencies. The (trainee) teachers from Cluster 2 seem to have the most balanced mix in terms of behavioral flexibility, dynamics, and resistance. As our analysis shows, the resilience competence “dynamism,” as an equilibration regulative and a competence to initiate changes as soon as they are necessary or desirable and to abandon time-honored habits or practices, showed itself to be the strongest separation factor between clusters. On the one hand, this finding could indicate that the mentioned resilience competence is most important in teacher training; on the other hand, it could be a result of sample selection. Interestingly, the three manifestations of resilience competencies showed striking similarities to the study of Bowles and Arnup (2016) and a former study conducted by Bowles and Hattie (2013). Bowles and Arnup (2016, p. 147) investigated “the link between adaptive functioning and resilience in early career teachers.” The authors identified three typologies of adaptability—stabilizers, adapters, and innovators—and showed that resilience is strongly associated with these typologies of adaptive change. Their analysis showed that *stabilizers* were the least resilient, which corresponds to Cluster 1 in our study with the lowest level of resilience competencies. *Adapters* were more resilient, corresponding to Cluster 3 in our sample with an average level of resilience competencies. Finally, *innovators* were most resilient, corresponding to Cluster 2 with the strongest level of resilience competencies in our sample. According to Bowles and Arnup (2016), the close relation between resilience and adaptive functioning can also be found in other studies (Gu and Day, 2007; Mansfield et al., 2012). This leads us to assume that our 3-cluster solution of resilience competencies can be linked to the concept of adaptive change. What reinforces our suspicion is that neither in our study nor in the study conducted by Bowles and Arnup (2016), was length of service significantly associated with resilience.

Regarding our *second* hypothesis, we found significant correlations between some resilience competencies and occupational demands or stress appraisal, respectively. *Flexibility* was significantly negatively correlated with all occupational demands—that is, the higher the degree of flexibility, the less burdening occupational demands were perceived to be. Moreover, all resilience competencies were significantly negatively correlated with the *demands regarding working conditions and workload*. Our second hypothesis can therefore be partly confirmed.

Regarding our *third* hypothesis, *flexibility* was significantly negatively correlated with all stress symptoms, except for *physical stress symptoms*. *Flexibility* was further significantly positively correlated with *well-being, job satisfaction* and *job engagement*. This can be an indication that (trainee) teachers with a certain degree of behavioral flexibility do indeed show less stress symptoms or emotional exhaustion and not only feel better but also have a better work experience. These findings are in line with the results of Pretsch et al. (2012), who found significant positive correlations between teacher resilience and well-being as well as job satisfaction. The importance of flexibility with respect to (preservice) teacher resilience is also highlighted by Le Cornu (2009), Mansfield et al. (2012), and Tait (2008). Indications for our third hypothesis can also be found in *dynamism*, as *emotional stress symptoms (anxiety)*, and *emotional exhaustion* are significantly negatively and *work experience* significantly positively correlated with this resilience competence.

### Limitations of the Study

Our study has strengths and weaknesses that must be weighed against each other. We not only asked prospective teachers in training to participate in our survey but also fully trained teachers (mean vocational experience as a teacher of 11.13 years), who have already completed their practical teacher training to improve our response rate. A bias in their retrospective assessments with regard to the perceptions experienced during teacher training cannot be ruled out. However, none of the three resilience competencies were significantly correlated with sociodemographic variables. Moreover, no cluster differences regarding training status were found, both indicating that trainee teachers and already trained teachers are comparable concerning the assessed resilience competencies.

Bearing in mind the (trainee) teachers' experienced demands were assessed on a 4-point Likert-type scale (1 = “not demanding,” 2 = “little demanding,” 3 = “demanding,” and 4 = “very demanding”), the highest value was achieved for working conditions and workload (M = 3.01). Social conditions (*M* = 2.04) and training-related aspects (*M* = 2.26) were evaluated as less demanding (Table 2). As the survey was conducted on a voluntary basis, it cannot be ruled out that the sample is selective—that is, that mainly those persons who participated in the survey are (were) not under great stress during their traineeship. Further, the sample is not representative for other domains and vocations; thus, the findings are not generalizable to other contexts.

Concerning the interpretability of our findings, the correlative relationships are based on cross-sectional data. This means that no statements can be made about causal interpretations for the correlations found. Further, no statements about the assumed alterable nature of the described resilience competencies (i.e., in terms of learnability) can be made on the basis of cross-sectional data. Although we cannot draw any causal conclusions from our study, we were able to show that there are basically three manifestations of resilience competencies among VET (trainee) teachers in Germany, which are closely related to the concept of adaptive change (see Bowles and Hattie, 2013; Bowles and Arnup, 2016). In order to investigate causal relationships between resilience competencies and occupational stress in teacher training, as well as the development of resilience competencies in the course of time, a longitudinal study design or an experimental design is needed.

Considering that stress is not a one-, but a multidimensional phenomenon—as psychological and somatic processes constituting a complex system of relations are involved (e.g., Kemeny, 2003; Kärner et al., 2017; Friederichs et al., 2020, 2021)—we only used self-reports to measure participants' physiological symptoms. Thus, further investigations should also use somatic markers, such as those of the autonomic nervous system, to measure physiological responses to occupational stress in teacher training.

### Implications for VET Teacher Training and the Need for Systemic Equilibration

From our study findings we suggest the integration of certain stress-management programs or, in particular, resilience trainings into the teacher training process. Studies regarding such interventions in teacher training are rare. Nevertheless, Dicke et al. (2015) were able to show that their stress-management training in groups had positive effects on trainee teachers' well-being. The authors assume a relationship here, as the perception of higher classroom management skills led to lower perceived strain and higher perceived well-being, confirmed by studies conducted with teachers in general. In addition to stress-management training in face-to-face groups or self-help books, online stress-management training is playing an increasingly important role and offers great opportunities for stress reduction (Hillert et al., 2019). One example of such online-based trainings is the BRiTE^4^ (*Building Resilience in Teacher Education*) program, which is especially designed for pre-service teachers and developed to promote resilience [BRiTE (Building Resilience in Teacher Education), 2014–2020]. The benefit of this particular program is highlighted by Beltman et al. (2018) who found evidence that the usage of these online modules supports the promotion of resilience in pre-service teachers. Nevertheless, such programs especially for (trainee) teachers have found relatively little application to date (Hillert et al., 2019; Sappa and Barabasch, 2020).

In the present study, we took a person-centered view on resilience. However, since individuals are embedded in specific contexts and exposed to specific environments, further studies would need to include additional contextual resources. Such contextual resources in vocational schools include, for instance, intra-scholastic support services, a supportive school leadership, and a collaborative school climate (Boldrini et al., 2019; Sappa et al., 2021). In that regard, Sappa et al. (2021) stress the necessity of *multi-level interventions*. This also seems relevant because resilience is not only a characteristic of individuals, but also of social systems and organizations (e.g., Holling and Gunderson, 2002; Keck and Sakdapolrak, 2013). Crises or external shocks, such as distance education as a result of COVID-19-related school closures, are also accompanied by stress at the organizational level and require appropriate balancing. Institutions and their actors are challenged to recognize destabilizations of system-inherent relationships, rules and organizational structures caused by external and internal disturbances, and further, to ensure their restabilization in accordance with valid, participatively determined and generally accepted social and human values and norms (Reinke and Kärner, 2020). Thus, in a broader sense stress-management programs should take into account various *ontogenetic and sociogenetic stratifications* that include a societal (“macro”), group interactive (“meso”), individual (“micro”), and biological (“nano”) level. *Balancing and evaluation processes* play a central role on each of the mentioned stratifications. In this regard, evaluation means the affective appraisal of internal and external stimuli. Balancing stands for equilibrating regulatory processes between diametrically opposed or even antagonistic subject areas, e.g., free, chaotic-ideal and individual perspective vs. normative, ordered and social perspective (Sembill and Kärner, 2018, 2020).

In order to reach *systemic equilibration*, on each of the above-mentioned stratifications corresponding target variables for interventions must be identified and defined. On a biological (“nano”) stratification it is relevant to support an individuals' somatic adaptation to stress. This can be achieved by interventions that address parasympathetic activity (Friederichs et al., 2021), for instance, via biofeedback training that helps individuals to sensitize one's own body perception (Goessl et al., 2017). As results of Friederichs et al. (2021) show, somatic stress regulation is associated with behavioral dynamics in terms of an equilibration regulative for being able to move appropriately on the continuum between adaptation and resistance (Sembill and Kärner, 2018; Friederichs et al., 2021). In that regard, on an individual (“micro”) and on an interactive (“meso”) stratification, behavioral resilience competencies must be promoted in order to help individuals to perceive one's own and others' demands and needs, to reflectively but also critically engage with others, and to take responsibility for self and others (Sembill and Kärner, 2018, 2020; Kärner and Sembill, 2021). On a societal (“macro”) stratification, the actors of the system, such as (trainee) teachers in our consideration, need to be empowered to actively participate in and shape risky development processes in changing personal and social environments and to develop a sense of distributive justice and sustainable conservation of personal and societal resources. On an *individual level*, this requires not least the development of the ability to counter the power of the supposedly factual with a creative “will to not have to” (Sembill, 1995; Kärner and Sembill, 2021). Our results support this assumption because behavioral dynamics, as equilibration regulative, apparently discriminates very well within specific groups of (trainee) teachers. One might speculate that evaluating specific trainings that influence this competency might be useful. On an *educational and systemic level*, this requires not least a discourse about underlying conceptions of man. On the one hand, humans can be regarded as beings who are responsible for their own actions and carry them out in a self-determined manner, as well as critically questioning and reflecting on external requirements. On the other hand, humans can also be seen as beings who uncritically accept what they are given externally (Heid, 2003, 2018). Future research efforts should therefore address the question of the concrete design of interventions at different ontogenetic and sociogenetic stratifications. This seems relevant because the targeted promotion of individual resilience competencies should ultimately lead to humane and health-preserving learning and working conditions.

## Data Availability Statement

The datasets presented in this article are not readily available because the data will not be shared publicly because for data protection reasons. Requests to access the datasets should be directed to tobias.kaerner@uni-hohenheim.de.

## Ethics Statement

Ethical review and approval was not required for the study on human participants in accordance with the local legislation and institutional requirements. The patients/participants provided their written informed consent to participate in this study.

## Author Contributions

TK: conceptualization, methodology, software, formal analysis, data curation, writing–original draft, writing–review, editing, visualization, supervision, and project administration. MB: conceptualization, methodology, formal analysis, writing–original draft, writing–review, and editing. EF and DS: writing–original draft, writing–review, editing, and validation. All authors contributed to the article and approved the submitted version.

## Conflict of Interest

The authors declare that the research was conducted in the absence of any commercial or financial relationships that could be construed as a potential conflict of interest.

## Footnotes

^1^In the following, the term “seminar” is used as a synonym for the *colleges of didactics and teacher education*, the place (outside of the training schools) where teacher training takes place.

^2^Kärner, T., Goller, M., Bonnes, C., and Maué, E. (in revision). Die professionelle pädagogische Beziehung zwischen Referendar*innen und ihren Seminarlehrkräften: Belastungsfaktor oder Ressource? [The professional pedagogical relationship between trainee teachers and their seminar mentors: stress factor or resource?].

^3^Training-related questions also had to be answered by the fully trained VET teachers, based on their memory.

^4^https://www.brite.edu.au/.
